# A Mystery in a Case: Unraveling the Complexity of Bing-Neel Syndrome

**DOI:** 10.7759/cureus.65042

**Published:** 2024-07-21

**Authors:** Payton N Kotlarz, Gustavo Garcia, Manuel Rosario, Ian Vargas, Kevin Cai Zhen

**Affiliations:** 1 Internal Medicine, Florida State University College of Medicine, Tallahassee, USA; 2 Internal Medicine, Florida State University College of Medicine, Sarasota Memorial Hospital, Sarasota, USA

**Keywords:** waldenström’s macroglobulinemia, ibrutinib therapy, central nervous system metastasis, myd88 mutation, lymphoplasmacytic lymphoma, bing-neel syndrome

## Abstract

Waldenström’s macroglobulinemia (WM) is a B-cell non-Hodgkin’s lymphoma characterized by clonal IgM-secreting lymphoplasmacytic cell proliferation. Bing-Neel syndrome (BNS) is a rare complication of WM that results in the infiltration of the central nervous system (CNS) with IgM-secreting lymphoplasmacytic cells. This case study presents a 75-year-old Caucasian male with a history of WM and Agent Orange exposure who ultimately was diagnosed with BNS. This patient posed unique diagnostic challenges as the patient experienced clinical symptoms despite the absence of MRI abnormalities and therapeutic challenges.

## Introduction

Waldenström’s macroglobulinemia (WM) is classified as a B-cell non-Hodgkin’s lymphoma that results in clonal IgM-secreting lymphoplasmacytic cell proliferation primarily in the bone marrow [1,2]. First identified by Jens Bing and Axel Valdemar Neel in 1936, Bing-Neel syndrome (BNS) is a rare clinicopathological complication of WM that results from the direct involvement of the central nervous system (CNS) with IgM-secreting lymphoplasmacytic cells [1-4]. Though the exact incidence is unknown, a relative frequency of 0.8% has been reported [2]. The diagnostic challenge of BNS arises from the diverse spectrum of symptoms, limited research, and the lack of a classical clinical vignette [3]. Our case underscores the complexity of diagnosing BNS and highlights the importance of upholding a heightened clinical suspicion in patients diagnosed with WM and unexplained neurological symptoms. This article was previously presented as a meeting abstract at the Florida Chapter American College of Physicians Medical Students Spring Poster Competition on March 23, 2024, and the American College of Physicians National Abstract Competition on April 20, 2024. 

## Case presentation

**Figure 1:**
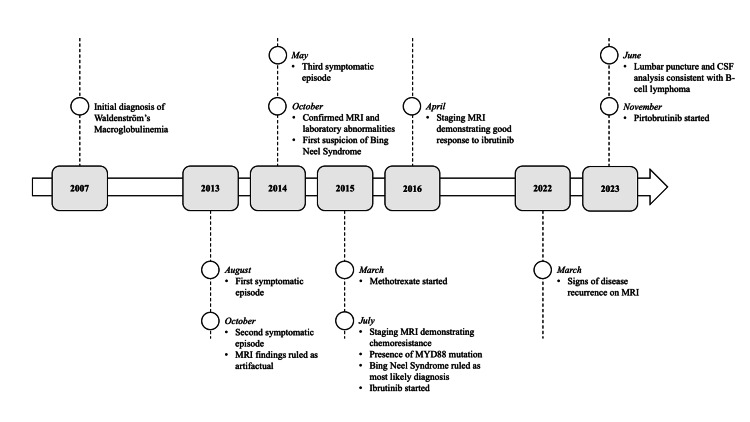
Key events in the patient’s diagnosis and treatment of Bing-Neel syndrome from 2007 to 2023

This report details the case of a 75-year-old Caucasian male with a past medical history significant for prostate cancer, diverticulitis, dyslipidemia, essential hypertension, Agent Orange exposure, and a six-year history of WM. Initially diagnosed with WM in 2007 (Figure 1), he was successfully treated with rituximab, cyclophosphamide, vincristine, and prednisone, followed by two years of maintenance rituximab, and remained asymptomatic for four years.

Initial Presentation

In August 2013, the patient presented to the emergency department (ED) with complaints of “lightheadedness, balance issues, and feeling weird.” These symptoms began a month prior, characterized by spells of lightheadedness and a sensation of warmth in his cervical and occipital regions. However, over the last two weeks, the frequency of his episodes had increased, prompting further investigation. Upon admission, a physical exam revealed slight gait abnormalities and difficulty with tandem walking. Laboratory analysis demonstrated elevated serum globulin levels. CT, MRI, carotid Doppler, and electroencephalogram (EEG) did not demonstrate abnormalities. He was discharged with a suspected eight nerve dysfunction or vestibular pathology with the possibility of a complex partial seizure and recommended to follow up with a neurologist.

Subsequent Developments

Two months later, the patient returned to the ED with language difficulties and dominant-handed clumsiness. A physical exam showed mild anomia, phonemic paraphasias, difficulty with mathematics, and delayed finger-to-nose testing on his dominant side. Complete blood count (CBC), coagulation factors, cultures, electrolytes, and liver functions were within range. He had mildly elevated serum globulin levels at 4.4 g/dL. CT imaging and carotid Doppler demonstrated no abnormalities, but an MRI showed subtle abnormal signals in the sulci at the vertex, considered likely artifactual but possibly indicative of infectious or neoplastic leptomeningeal disease. Neurology found no abnormalities correlating with the patient’s symptoms, and a complicated migraine was deemed most consistent with his presentation. As a precaution, he was placed on clopidogrel, atorvastatin, and amlodipine and advised to follow up with neurology.

Third Presentation and Initial Suspicions 

In May 2014, the patient presented with “left upper extremity tingling and mild difficulty with speech,” which resolved without any residual deficits. Further evaluation demonstrated no abnormalities. Given his WM history and a potential hypercoagulable state, a transient ischemic attack (TIA) was identified as the most likely diagnosis. He had plans to follow up with his hematologist-oncologist, neurologist, and primary care physician upon discharge.

**Figure 2:**
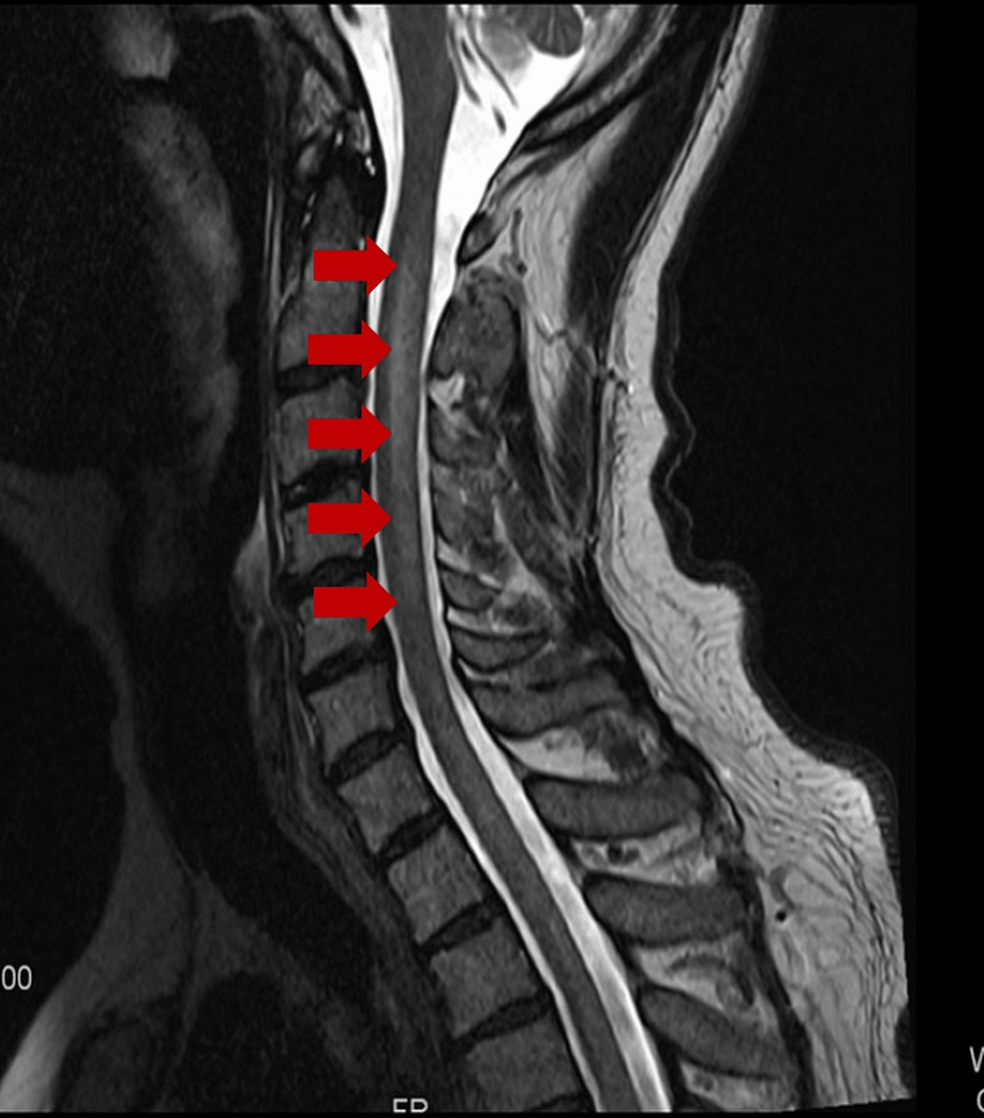
Leptomeningeal enhancement along the cervical cord

Five months later, an MRI with gadobenate dimeglumine indicated subtle parenchymal enhancement in the left frontal white matter, left parietal lobe, and likely anterior temporal lobe regions, with ventricular wall enhancement. Laboratory analysis revealed cerebrospinal fluid (CSF) protein levels at 190 g/dL. These findings, along with his gait abnormalities and fatigue, led to a lumbar punction (LP) and CSF flow-cytometry, revealing small B-cells (20% of total cells) with lambda restriction, mirroring that of the bone marrow (Table 1). An MRI with gadobenate dimeglumine demonstrated intramedullary and leptomeningeal enhancement along the cervical cord, brainstem, and cerebellum (Figure 2). The patient was diagnosed with a malignant neoplasm of cerebral meninges, strongly suggestive of BNS.

**Table 1:** The patient's CSF flow cytometry results CSF: Cerebrospinal fluid

Date	% Lymphocyte	Cell Type	Immunophenotype	Kappa:Lambda Ratio	CD4:CD8 Ratio
12/10/2014	25	B-lymphocytes	CD19+, CD5-, CD10-	0.01 (Clonal)	
	66	T-lymphocytes (CD3+CD5+)	CD3+CD5+		0.8 (Skewed/Inverted)
1/14/2015	19	B-lymphocytes	CD19+CD20+	0.01 (Clonal)	
	74	T-lymphocytes (CD3+CD5+)	CD2+CD3+CD5+CD7+		1.2
	5	Natural Killer Cells	CD56+CD2+CD7+CD3-		
Date	% Total Cells	Cell Type	Immunophenotype		
12/10/2014	79	Lymphocytes	Strong CD45+		
	15	Myeloid			
	3	Monocytes			
	3	CD45-			
1/14/2015	88	Lymphocytes	Strong CD45+		
	< 0.1	Myeloid			
	10	Monocytes			
	< 0.1	CD45-			
	< 0.1	CD45 dim			
	0.15	Myeloblast	CD34+		

Diagnosis, Treatment, and Management

A repeat LP and CSF flow cytometry in January 2015 demonstrated an abnormal population of 19% lymphocytes with an immunophenotype of CD19+, CD20+, CD38+, CD5-, CD10-, CD23-, and positive monoclonal lambda light chain (Table 1). In March 2015, he began high-dose methotrexate and rituximab infusions. Despite complications, including generalized seizures due to delayed methotrexate clearance, he completed eight cycles with good tolerance. Four months later, a staging MRI showed a presacral mass and left-sided mild hyperintensities in the frontal periventricular white matter, parietal subcortical white matter, and posterior ventricular wall. Additionally, subtle supratentorial and infratentorial leptomeningeal enhancement was observed, primarily involving the basal ganglia and bilateral cerebral hemispheres on T2-weighted fluid-attenuated inversion recovery (T2-FLAIR) imaging. CSF analysis revealed an elevated protein level of 214 mg/dL, while LP demonstrated a myeloid differentiation factor 88 gene (MYD88) mutation. The presence of an MYD88 mutation, in combination with the patient’s MRI results, CSF analysis, and chemoresistance, strongly supported a diagnosis of BNS.

The patient began ibrutinib therapy at 420 mg daily, resulting in a significant clinical response as demonstrated by improved gait, memory, and fatigue. New MRI staging in April 2016 showed decreased leptomeningeal enhancement and presacral mass size. Laboratory analysis revealed a significant reduction of monoclonal cells and CSF protein, while serum protein electrophoresis (SPEP) demonstrated decreased IgM levels. 

Recent Developments

**Figure 3:**
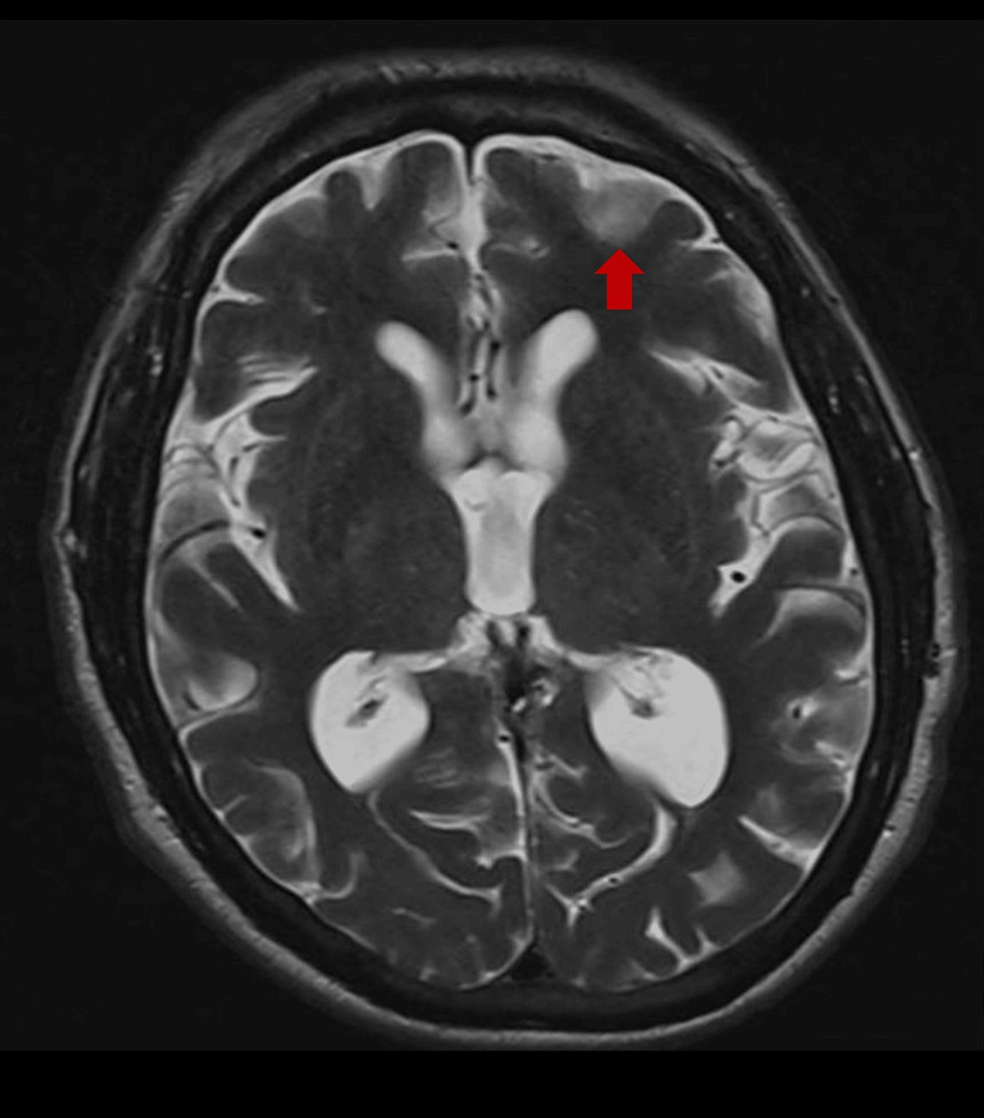
Enhancement of a deep left frontal lesion on T2-FLAIR imaging T2-FLAIR: T2-weighted fluid-attenuated inversion recovery

In March 2022, he presented with word-finding difficulties, generalized weakness, and gait imbalance following a tixagevimab/cilgavimab injection. He was initially diagnosed with toxic-metabolic encephalopathy but returned the following day without resolution of symptoms. His SPEP confirmed the presence of monoclonal proteins. IgA, IgG, and IgM levels were within normal limits, and his Kappa:Lambda ratio was 1.35. An MRI with gadobenate dimeglumine revealed moderate global atrophy and a deep left frontal lesion on T2-FLAIR imaging (Figure 3). LP without flow cytometry demonstrated a total protein level of 156 mg/dL, elevated WBC at 24 cells/μL, negative cytology, and no evidence of malignant cells. He eventually recovered without the need for intervention.

In May 2023, he presented with speech changes, lethargy, and confusion. CT imaging demonstrated no abnormalities, and his MRI was unchanged from March 2022. One month later, LP with CSF analysis demonstrated atypical lymphocytes with flow cytometry revealing the presence of CD5-, CD10-, and CD20+ B-cells consistent with low-grade B-cell lymphoma. Subsequently, he transitioned from ibrutinib to pirtobrutinib, but due to difficulties in obtaining pirtobrutinib, he continued treatment with ibrutinib at 420 mg daily, with the addition of venetoclax at 100 mg daily. However, he discontinued venetoclax following several episodes of severe vertigo and vomiting after a 100 mg increase in his dosage.

Current Status

**Figure 4:**
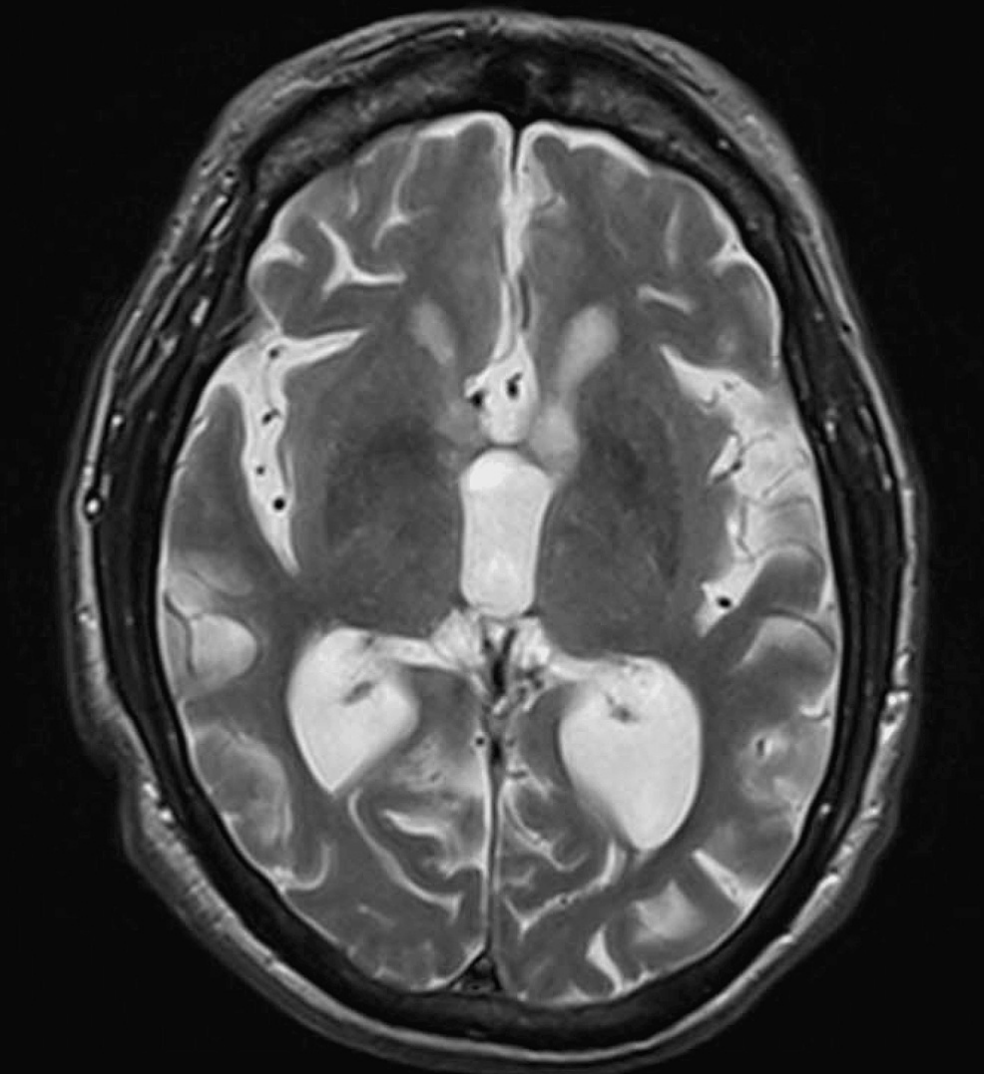
MRI illustrating no acute hyperintensities or lesions on T2-FLAIR imaging following treatment with pirtobrutinib T2-FLAIR: T2-weighted fluid-attenuated inversion recovery

Five months later, the patient discontinued ibrutinib and began pirtobrutinib, showing clinical improvement and decreased M-protein spike and total protein levels. MRI demonstrated no acute hyperintensities or lesions on T2-FLAIR imaging (Figure 4). He continues to be monitored, demonstrating a positive response to the treatment.

## Discussion

BNS is a rare complication of WM that results in the direct involvement of the CNS with IgM-secreting lymphoplasmacytic cells [1,2,4]. Diagnosing BNS is particularly challenging, given that the clinical presentation varies depending on which regions of the brain are affected by the infiltration of lymphoplasmacytic cells. A comprehensive review of the two largest retrospective studies available examined the symptomatology of 78 patients with BNS [5]. Their findings revealed a variety of symptoms, with 48% of patients presenting with balance disorders or ataxia, 36% with cranial nerve involvement, 27% with cognitive impairment, 25% with motor or sensory involvement, and 18% with headaches [5]. Other manifestations included cauda equina syndrome, visual impairments, hearing deficits, and psychiatric symptoms [5].

Furthermore, diagnosing BNS poses a considerable challenge due to the overlapping symptomatology with other disease processes. For instance, hyperviscosity syndrome, characterized by increased blood viscosity resulting from pathological elevations in RBC, WBC, serum proteins, or platelets, or deformities in RBC shape, can lead to decreased cerebral circulation [6]. This condition often arises secondary to WM and typically manifests with visual disturbances such as blurry or cloudy vision, mucosal bleeding, and neurological symptoms mirroring those observed in BNS [6]. Additionally, meningitis, inflammation of the meninges, and encephalitis, inflammation of the brain itself, may present with symptoms like those in BNS, particularly in patients with underlying malignancies or immunosuppression [7]. Focal impaired awareness seizures, also known as complex partial seizures, begin in one cerebral hemisphere and yield various neurological symptoms contingent upon the affected brain region [8]. Despite being the most common cause of adult epilepsy, complex partial seizures can present challenges in diagnosis as EEG findings may remain unremarkable even during active episodes due to subcortical electrical charges [9]. In our patient’s case, suspicion of these differential diagnoses prompted thorough laboratory tests and imaging studies, contributing to the delayed diagnosis.

The diagnosis of BNS relies on a comprehensive diagnostic approach beginning with an MRI of the brain and spinal cord [3,4,10]. MRI findings can vary, but common radiologic findings include enhancement of the leptomeningeal sheaths and perivascular spaces in the diffuse form or involvement of the deep subcortical hemispheric regions in the tumoral form [3]. As illustrated in this case, our patient presented with symptoms, yet initial imaging failed to reveal disease manifestations. A similar phenomenon occurred in a recent retrospective review where all diagnoses of BNS were confirmed by CSF analysis since most of their patients had nonspecific radiological findings [11]. Although the exact reasons for absent or nonspecific MRI signals in BNS patients are uncertain, several possible explanations exist. First, in the early stages of BNS, the extent of leptomeningeal infiltration might be minimal, making detectable changes on MRI unlikely and leading to inconclusive results. Early or small lesions may also be challenging to distinguish from normal brain tissue in the early disease stages if advanced imaging techniques or targeted MRI sequences are not employed. Additionally, the initial imaging might not have focused on the specific areas of the brain and spinal cord where early leptomeningeal enhancement occurred. Lastly, the intermittent nature of the patient’s presentation may suggest that the pathological changes were not constant or widespread enough to be captured on initial imaging.

MRI of the brain and spinal cord is then followed by flow cytometric analysis of the CSF with routine leukocyte counts and biochemistry panels [3,4,10]. Histological biopsy of the meninges or cerebrum demonstrating lymphoplasmacytic lymphoma is currently considered the gold standard [3-5]. Flow cytometry typically reveals monotypic B-cells expressing pan B-cell antigens CD19, CD20, CD79a, and CD79b, mirroring that of WM [3,5]. While not mandatory, the detection of a somatic mutation in the MYD88 gene, resulting in the substitution of a leucine with a proline at position 265, on immunoglobulin gene rearrangement analysis of the CSF strongly supports the diagnosis, as demonstrated in our case [3,10]. The MYD88 gene, prevalent in B-cell lymphomas and primary CNS lymphomas, serves as a crucial adaptor protein in interleukin-1 (IL-1) and Toll-like receptors (TLRs) signaling pathways and in the activation of nuclear factor kappa-light-chain-enhancer of activated B-cells (NF-kB), and Janus kinase/signal transducers and activators of transcription (JAK/STAT) pathways [12-14]. Notably, a gain-of-function mutation in the MYD88 gene leads to dysregulated inflammation, tissue damage, apoptosis inhibition, and upregulation of pro-survival genes [15]. The average time to diagnosis in patients with BNS as their first manifestation of WM varies from four to 36 months [16]. In patients with a history of WM, the time to BNS diagnosis varies across literature, ranging from 3.5 to seven years [11,17]. 

Current treatment options for BNS include several approaches: multiple cycles of high-dose methotrexate or high-dose cytarabine; standard doses of fludarabine, cladribine, or bendamustine; and daily doses of ibrutinib at either 420 mg or 560 mg [3]. Although drugs like methotrexate have traditionally been employed in treating lymphoplasmacytic lymphomas due to their cytotoxic properties and ability to cross the blood-brain barrier, recent support has emerged for ibrutinib, a Bruton tyrosine kinase inhibitor. This transition is attributed to its targeted mechanism, which involves downregulating B-cell receptor signaling (BCR) and modulating tumor microenvironments (TME), ultimately inducing apoptosis and controlling lymphocytosis [18].

In a single-arm, multicenter study involving 106 patients with treatment-naïve or relapsed/refractory WM, treatment with acalabrutinib demonstrated promising response rates of 93% and 95%, respectively [19]. Notably, after 66 months, the progression-free survival was 84% in the treatment-naïve group and 52% in the relapse/refractory group [19]. Furthermore, in a retrospective review involving 28 patients, ibrutinib was administered orally, once daily, with doses of 560 mg in 46% of patients and 420 mg in 54% of patients [17]. Most patients experienced an improvement or resolution of BNS symptoms and radiological abnormalities (85% and 83%, respectively) at maximum response [17]. Despite these promising findings, our patient experienced symptomatic improvement initially with ibrutinib but unfortunately relapsed. The lack of a standardized treatment approach underscores the ongoing challenge of managing BNS effectively.

The survival rate of patients with BNS has been reported across the literature to be 81% at two years with ibrutinib treatment, 71% to 86% at five years, and 59% at 10 years post-diagnosis. However, due to the low prevalence of BNS, definitive prognosis data for patients with and without treatment remains largely unknown [5,16,17].

## Conclusions

As demonstrated, the nonspecific and diverse symptoms associated with BNS present a complex diagnostic challenge, with no single clinical presentation including or excluding the diagnosis. This case posed unique challenges, as the patient experienced clinical symptoms despite the absence of MRI abnormalities. Consequently, it is crucial to maintain a high index of suspicion for BNS in patients with a history of WM who present with neurological symptoms. Several lessons from this case can help to improve the early detection of BNS. These lessons include performing detailed and repeated neurological examinations, using targeted and sequenced MRIs, utilizing early genetic testing, and employing a multidisciplinary care team. Such an approach is essential for preventing diagnostic oversights and improving the quality of life for affected patients.

Given the rarity of BNS, available data is predominantly derived from case reports and small center studies, posing challenges in standardizing diagnosis and treatment. Future research may focus on addressing these challenges by incorporating the following: long-term follow-up using patient-reported outcomes; optimizing treatment strategies through the exploration of emerging Bruton tyrosine kinase inhibitors and monoclonal antibodies; investigating mechanisms of CNS infiltration, neuroinflammation, and disruption of the blood-brain barrier; and assessing genetic predisposition and environmental factors contributing to disease development.
